# Clinical and Laboratory Characteristics of Neonates Treated Due to Suspected Serious Bacterial Infection: Single Center Cross-Sectional Study

**DOI:** 10.3390/pediatric17050107

**Published:** 2025-10-14

**Authors:** Klara Rezic, Ivan Simunovic, Hrvoje Saric, Josko Markic

**Affiliations:** 1 School of Medicine, University of Split, 21000 Split, Croatia; klara.rezic@gmail.com (K.R.); ivan.simunovic99@gmail.com (I.S.); 2 School of Dental Medicine, University of Zagreb, 10000 Zagreb, Croatia; hrvoje.saric9@gmail.com; 3 Department of Pediatrics, University Hospital of Split, 21000 Split, Croatia

**Keywords:** serious bacterial infection, neonates, urinary tract infection, C-reactive protein

## Abstract

**Background**: Serious bacterial infections (SBIs) in neonates present a significant diagnostic challenge due to nonspecific symptoms and immature immune responses. Early identification is essential to ensure timely treatment and prevent adverse outcomes. This study investigates clinical, laboratory, and epidemiological parameters associated with SBI in febrile neonates. **Methods**: A retrospective analysis was conducted on neonates hospitalized for suspected SBI at University Hospital Split from 1 January 2023 until 31 December 2024). The data was analyzed using descriptive statistics, Mann–Whitney U test and Chi-square test. **Results**: The study included 71 neonates hospitalized with suspected SBI, of whom 38 (53.5%) had a confirmed SBI. Neonates with SBI had a significantly longer hospital stay (*p* < 0.001). C-reactive protein (CRP) levels at admission were significantly higher in the SBI group (*p* = 0.020), while other laboratory parameters showed no significant differences. The most common diagnosis in the SBI group was urinary tract infection (50%). In urine analysis, abundant bacterial presence, strongly positive leukocyte esterase (3+), and positive nitrite findings were significantly associated with the presence of urinary tract infection (UTI). **Conclusions**: In neonates with suspected SBI, elevated CRP levels and prolonged hospital stay were significantly associated with confirmed SBI. Among specific diagnoses, UTI were most frequent, with urinalysis parameters (bacteria, leukocyte esterase, and nitrites) proving useful in identifying affected cases. However, individual clinical signs showed limited diagnostic value, highlighting the importance of combining clinical and laboratory data in early recognition of SBI.

## 1. Introduction

Fever is one of the most common reasons for emergency department visits among neonates and young infants. In the vast majority of cases, it is caused by mild, self-limiting viral infections that do not require hospitalization [1]. Nevertheless, neonates represent an extremely vulnerable population due to the immaturity of their immune system and their increased susceptibility to rapid progression of serious bacterial infections [2].

Serious bacterial infection (SBI) is defined as the presence of one or more of the following conditions: bacteremia, bacterial meningitis, sepsis, pneumonia, urinary tract infection (UTI), bacterial gastroenteritis, or cellulitis [3].

The overall prevalence of SBI in neonates ranges from 5% to 20%, with UTI being the most frequently identified type [2,3,4].

The diagnostic evaluation of SBI in neonates and infants begins with a comprehensive clinical examination. This patient population presents unique challenges, as the clinical manifestations of SBI are often nonspecific. The general condition of the neonate may appear normal, and fever, commonly the only indicator of infection, may be absent. A detailed clinical assessment is essential and should include systematic observation, evaluation of vital signs, and a detailed examination of all organ systems. Observational findings that may suggest SBI include lethargy, reduced spontaneous motor activity, somnolence, weak or high-pitched crying, peripheral cyanosis or pallor, a grayish or pale facial complexion, and mottled skin [5]. Clinical indicators of severe illness in infants are diverse and frequently involve multiple organ systems. Dermatological signs such as petechiae may serve as early indicators of systemic infection. Neurological symptoms can include pronounced drowsiness alternating with irritability, seizures, and a bulging fontanelle, particularly in cases of meningitis, where classical meningeal signs are often absent [6]. Gastrointestinal manifestations commonly include decreased oral intake, frequent vomiting, abdominal distension, and diarrhea [7]. Respiratory abnormalities may present as apnea or tachypnea, often accompanied by use of accessory muscles, such as intercostal and suprasternal retractions [5]. Indicators suggestive of circulatory compromise or shock include mottled skin, prolonged capillary refill time, cyanosis, and oliguria [8].

In addition to the nonspecific clinical presentation, the diagnosis of SBI in neonates is further complicated by the limited reliability of laboratory tests in this age group. No single parameter can reliably exclude SBI, often leading to empirical antibiotic treatment and hospitalization of all febrile neonates [9,10]. Laboratory evaluation typically includes a complete blood count (CBC) with differential, total leukocyte count, and microbiological cultures, particularly blood and urine cultures. The absolute neutrophil count (ANC) is commonly used to assess infection risk, but its diagnostic accuracy, along with that of white blood cell (WBC) count, is limited in neonates [11]. Due to these limitations, interest has shifted toward alternative biomarkers such as procalcitonin (PCT) and C-reactive protein (CRP), which have shown promise in differentiating bacterial from viral infections [12]. Nonetheless, accurate early diagnosis of SBI remains challenging and requires integration of clinical findings with laboratory and microbiological data.

Although SBI is ultimately confirmed in only a minority of cases (5–15%), early recognition and timely treatment are critical to preventing severe complications [2,13]. At the same time, identifying infants at low risk for SBI is essential to avoid unnecessary antibiotic use and hospitalization, thereby reducing the risk of antimicrobial resistance, toxic and allergic reactions, nosocomial infections, and the financial and logistical burden on both the healthcare system and families [14].

This study aimed to analyze the clinical, laboratory, and epidemiological characteristics of neonates hospitalized due to suspected SBI. Additionally, it intended to identify potential differences in clinical presentation and laboratory parameters between neonates with or without confirmed SBI. The findings contribute to a better understanding of diagnostic challenges in this vulnerable population and emphasize the need for more accurate risk stratification and management strategies.

## 2. Materials and Methods

### 2.1. Study Design

This retrospective observational study included neonates aged 0 to 28 days presenting to the pediatric emergency department who were subsequently hospitalized at the Department of Pediatrics, University Hospital Center Split, from 1 January 2023, to 31 December 2024, due to suspected SBI. Exclusion criteria were a confirmed viral infection identified by rapid antigen testing (influenza A/B, respiratory syncytial virus, SARS-CoV-2, adenovirus) and transfers from other healthcare institutions. Participants were divided into two groups based on discharge diagnoses: the SBI group (sepsis/bacteremia, bacterial meningitis, bacterial pneumonia, urinary tract infection, bacterial gastroenteritis, cellulitis) and the non-SBI group (all other diagnoses). The non-SBI group included infants with a variety of presumed viral or non-bacterial diagnoses. Data were collected retrospectively from medical records and included demographic characteristics (age, sex, body weight, body length), clinical presentation (body temperature and symptoms at admission), and laboratory parameters obtained at the pediatric emergency department at admission. Laboratory data comprised complete blood count (CBC), differential white blood cell count, and a panel of serum biochemical markers including C-reactive protein (CRP), glucose, calcium, potassium, chloride, creatinine, lactate dehydrogenase (LDH), magnesium, sodium, uric acid, and urea, as well as urinalysis. The duration of hospitalization was also recorded. The study was approved by the Ethics Committee of the University Hospital Center Split (Approval No. 520-03/25-01/05, dated 31 January 2025) and conducted in accordance with national regulations and international ethical standards.

### 2.2. Statistical Analysis

Statistical analysis was conducted using the R programming language (version 4.4.3), a specialized software environment for statistical computing and graphical data presentation. The Shapiro–Wilk test was applied to assess the normality of data distribution. Descriptive statistics for numerical variables were expressed as means and standard deviations for normally distributed data, and as medians with interquartile ranges for non-normally distributed data. To compare differences between the two independent groups (SBI and non-SBI), the following statistical tests were employed: Student’s *t*-test for normally distributed variables (e.g., body weight, length of hospital stay), the Mann–Whitney U test for non-normally distributed variables (e.g., C-reactive protein levels, leukocyte count), and the chi-square (χ^2^) test for categorical variables (e.g., sex, presence of specific symptoms or urine findings). A two-tailed *p*-value of less than 0.05 was considered statistically significant.

## 3. Results

A total of 71 neonates were included in the study. Participants were divided into two groups based on discharge diagnoses: the SBI group (*N* = 38) and the non-SBI group (*N* = 33). The SBI group consisted of 31 male and 7 female neonates, while the non-SBI group included 22 male and 11 female neonates. The most frequent diagnoses in the non-SBI group were unspecified viral infection (*N* = 10), upper respiratory tract infection (*N* = 6), bronchiolitis (*N* = 4), feeding difficulties (*N* = 3), neonatal jaundice (*N* = 2), and acute viral gastroenteritis (*N* = 2). There was no statistically significant difference in sex distribution between the groups (*p*-value = 0.243).

Table 1 presents the descriptive characteristics of the neonatal cohort. No significant difference was observed between the groups in terms of age at hospital admission, as well regarding mean body temperatures, body weight or body length. The only variable that showed a statistically significant difference between the groups was the length of hospitalization. Neonates in the SBI group had a significantly longer hospital stay (7.47 ± 3.06 days) compared to those in the non-SBI group (4.58 ± 2.81 days), (*p* < 0.001).

The distribution of clinical diagnoses within the SBI group is presented in Table 2. The most common diagnosis was UTI, recorded in half of the neonates with SBI (*N* = 19; 50.0%).

Table 3 presents the values of the analyzed laboratory parameters. Most hematological markers did not show statistically significant differences between the two groups. The red blood cell (RBC) counts were similar between the groups, as were hematocrit levels and hemoglobin concentrations. The white blood cell (WBC) count was elevated in the SBI group (median: 13.0 × 10^9^/L) compared to the non-SBI group (11.5 × 10^9^/L); however, the difference did not reach statistical significance (*p* = 0.209). Similarly, no significant differences were observed in the absolute neutrophil count (ANC) (*p* = 0.062), neutrophil percentage (*p* = 0.098), or lymphocyte percentage (*p* = 0.339). The mean corpuscular hemoglobin (MCH) and mean corpuscular hemoglobin concentration (MCHC) were also comparable between groups. The mean corpuscular volume (MCV) was slightly higher in the non-SBI group, but without statistical significance (*p* = 0.059). Platelet counts were similar between the groups.

Table 4 presents the values of the analyzed laboratory parameters. A statistically significant difference was found in CRP values, with participants in the SBI group having higher CRP levels (median 9.5 mg/L) compared to the non-SBI group (median 2.2 mg/L), with a *p*-value of 0.020. For the remaining analyzed laboratory indicators, no statistically significant differences were observed between the groups (all *p* > 0.05).

In 50% of neonates with SBI and 51.5% of those without SBI, no identifiable focal source of infection was observed. The most common clinical finding at admission was fever, observed in 57.9% of neonates with SBI and in 42.4% of those without SBI (Table 5). Although some clinical features appeared more frequently in the SBI group, such as tachycardia (28.9% vs. 15.2%) and mottled skin (13.2% vs. 3.0%)—these differences were not statistically significant. Poor general condition was equally represented in both groups (SBI: 15.8%; non-SBI: 15.2%), as were respiratory difficulties (15.8% vs. 21.2%). Other symptoms, including irritability, prolonged capillary refill time (CRT > 3 s), weak cry, and upper respiratory tract symptoms (pharyngeal catarrh, nasal congestion, nasal discharge), were rarely observed and did not differ significantly between groups. Thus, although certain clinical features were more common in neonates with SBI, the differences did not reach statistical significance, indicating the limited diagnostic value of individual symptoms in distinguishing between neonates with and without SBI.

Table 6 presents the distribution of key urinalysis findings among neonates with confirmed UTI. Majority in this group, as well in non-UTI SBI group were males (17/19, 89.5% vs. 14/19, 73.7%), respectively. All UTI patients demonstrated the presence of abundant bacteria in their urine samples. For leukocyte esterase, the majority (84%) showed a strong positive reaction (3+), 5% had a moderate reaction (2+), none had a mild positive result (1+), and 11% tested negative. Regarding nitrites, 63% of samples were positive.

## 4. Discussion

Neonates present a particular challenge for pediatricians due to their immature immune systems, which make them highly susceptible to developing severe clinical conditions following infection. Additionally, they often exhibit non-specific symptoms of infection, making it difficult to distinguish between those with SBI and those with a benign viral illness. A further complication is the lack of any laboratory test that can definitively confirm or rule out SBI. Consequently, all neonates with suspected SBI are frequently hospitalized to avoid missing treatment opportunities for those who are truly infected.

Among the 71 neonates hospitalized at the University hospital of Split between 2023 and 2024 due to suspected SBI, 38 cases (54%) were confirmed, while 33 (46%) were ruled out. The most commonly identified type of SBI in the study population was UTI, which aligns with findings from the available literature [15,16].

Our study found no statistically significant difference in gender distribution. This parameter remains a point of contention among previous studies. Several studies report that male neonates are at higher risk of SBI due to a significantly greater susceptibility to UTIs during the first month of life [4,17]. Conversely, many authors have not found any association between gender and the incidence of SBI [15,18]. These discrepancies may come from differences in ethnic backgrounds of the study populations. In populations where circumcision is routinely performed in male infants, UTI rates are significantly lower, which may contribute to the inconsistent results [8]. This is a parameter whose association with SBI should be better explored in future research. Although male circumcision is not routinely performed in our population, the results may also reflect the relatively small sample size.

A statistically significant difference was observed in the length of hospitalization, which was longer in neonates with confirmed SBI. This finding is expected and understandable, as per WHO guidelines, SBI treatment typically involves intravenous antibiotics for ten days in cases of sepsis and/or meningitis, and seven days for other types of SBI [19]. The hospital stay was shorter in the non-SBI group, as they did not require such intensive treatment. It is important to note that hospitalization itself carries a risk of nosocomial, multi-resistant infections, and thus, prolonged inpatient care should be reserved only for those neonates for whom the benefits clearly outweigh the risks [20,21].

Analysis of data presented in Table 6 indicates a clear association between positive findings of bacteria, leukocyte esterase, and nitrites in urine with the presence of UTI. Among UTI-positive subjects, there was a high prevalence of abundant bacteria (100%), strong positive leukocyte esterase (3+) in 84% of cases, and positive nitrite tests in 63% of cases. These results suggest that urine analysis, particularly findings of bacteria, leukocyte esterase and nitrite are highly indicative of UTI in this population. Thus, urine analysis represents a valuable diagnostic tool that aids in distinguishing UTI from other clinical conditions with similar symptoms, thereby increasing diagnostic accuracy and reducing the need for additional testing. Similar conclusions were drawn by Mahajan et al. in their 2022 study [22].

In our comparative analysis of laboratory markers between neonates with confirmed SBI and those in whom SBI was excluded, individual hematological parameters—such as WBC, ANC and lymphocyte count—did not show statistically significant differences. For example, although leukocyte levels were elevated in the SBI group compared to the non-SBI group, this difference was not statistically significant. However, a statistically significant difference was observed in CRP levels, with higher CRP values in the SBI group compared to the non-SBI group. This finding aligns with numerous previous studies that have recognized CRP as a more useful parameter for differentiating bacterial from non-bacterial causes of febrile illness in neonates [11,23]. Nonetheless, while CRP has greater diagnostic value than most other hematological markers, its specificity and sensitivity are still insufficient for reliably ruling out SBI [24,25]. Other studies have similarly concluded that standard laboratory tests, including CRP, lack the accuracy needed when used in isolation [10,26].

Our results further confirm the established clinical understanding that no single reliable laboratory test currently exists that can confidently exclude SBI in neonates. This remains a major challenge in therapeutic decision-making and underscores the need for the development of more accurate diagnostic tools in the future.

The clinical presentation of the neonates in this study confirms its highly non-specific nature, with significant overlap between infected and non-infected cases. Although certain signs, such as tachycardia or skin mottling, were somewhat more frequently observed in neonates with SBI, the differences were not statistically significant, highlighting the limited diagnostic value of individual clinical symptoms. These findings are consistent with numerous prior studies indicating that clinical signs of SBI in neonates are often absent or non-specific [27,28].

The results of this study further reinforce the notion that neither clinical presentation nor individual laboratory parameters are sufficiently reliable to differentiate SBI from benign, typically viral, conditions in neonates. SBI often presents subtly and non-specifically, with significant symptom overlap between neonates with SBI and those with viral infections; this was clearly reflected in our cohort. Similarly, most standard hematologic markers were not diagnostically distinctive, with CRP being the only one showing a statistically significant difference between groups. However, despite CRP being recognized in prior literature as a more useful biomarker, it still lacks sufficient specificity and sensitivity to be used independently to safely exclude SBI.

This study has several important limitations that should be considered when interpreting the results. First, it is a retrospective study conducted at a single center, limiting the generalizability of the findings. The sample size was relatively small, which further limits statistical power, especially in analyzing rare clinical signs or laboratory parameters and calculating the sensitivity and specificity of diagnostic tests. Second, clinical assessments upon admission were based on the subjective judgment of different physicians and possibly inconsistent symptom documentation. Additionally, due to the retrospective nature of the study, it was not possible to standardize the timing between symptom onset and laboratory testing, which may affect values of parameters such as CRP and leukocyte counts. Moreover, we lack data on how many neonates received antipyretics before arrival, which could blunt the clinical presentation, particularly fever. We believe the actual median body temperatures were likely higher than those recorded upon admission. Third, although some key laboratory markers were analyzed, not all potentially useful biomarkers were available, such as procalcitonin (PCT), presepsin, interleukin-6, or advanced parameters from automated blood analyzers (e.g., cell population data, CPD), which might offer additional diagnostic value. Finally, data on certain perinatal and maternal factors, such as prematurity status, mode of delivery, breastfeeding practices, and maternal colonization or infection were not available.

All these limitations highlight the need for future prospective, multicenter studies with larger sample sizes and broader diagnostic coverage to improve the reliability and applicability of findings in clinical practice. Given all the above, there is still no single clinical or laboratory test that can reliably exclude SBI in neonates. Therefore, physicians are often compelled to adopt a low threshold for hospitalization and empirical antibiotic therapy to reduce the risk of missing potentially life-threatening infections. As a result, a considerable number of neonates are unnecessarily exposed to hospital care and antibiotics, increasing the risk of nosocomial infections, antimicrobial resistance, and disruption of early microbiome colonization. This underscores the need to develop neonatal-specific, multicomponent diagnostic tools, identify new and more sensitive and specific laboratory markers for detecting SBI, and consider implementing available but costly infection biomarkers in clinical decision-making.

## 5. Conclusions

SBI in neonates remains a significant diagnostic and therapeutic challenge due to nonspecific clinical presentations and the limited reliability of individual laboratory markers. This study demonstrates that while CRP levels and prolonged hospital stays were significantly associated with confirmed SBIs, other hematological parameters and clinical signs lacked sufficient discriminatory power. However, these two findings must be interpreted differently. CRP represents a measurable biomarker with diagnostic value at the time of admission, whereas length of hospitalization is primarily determined by treatment needs and institutional protocols, and therefore reflects a clinical outcome. UTIs were the most common SBI, with urinalysis findings—abundant bacteria, strong leukocyte esterase, and positive nitrites—serving as reliable indicators. Given the limitations of current diagnostic tools, clinicians must continue to integrate multiple data points when evaluating febrile neonates. Further large-scale, prospective studies are essential to develop more accurate, neonatal-specific diagnostic algorithms and reduce unnecessary antibiotic use and hospitalization.

## Figures and Tables

**Table 1:** Characteristics of neonates upon admission to hospital.

	SBI (N = 38)	Non-SBI (N = 33)	*p*
**Age (in days)**	16.5 (10.75, 22)	17 (11, 24)	0.908 *
**Body temperature (°C)**	37.7 (36.7, 38.4)	37.4 (37, 38.4)	0.968 *
**Body weight (g)**	3812.50 ± 600.34	3774.85 ± 618.38	0.796 **
**Body length (cm)**	52.87 ± 2.59	53.24 ± 2.73	0.556 **
**Length of hospitalization (days)**	7.47 ± 3.06	4.58 ± 2.81	<0.001 **

The data are presented as median and interquartile range, as well as mean ± standard deviation. SBI = serious bacterial infection * Mann–Whitney U test. ** *t*-test.

**Table 2:** Frequency of specific types of SBI.

SBI	N	%
**UTI**	19	50.0%
**Pneumonia**	9	23.7%
**Gastroenteritis**	4	10.5%
**Cellulitis**	3	7.9%
**Sepsis**	2	5.3%
**Meningitis**	1	2.6%
**TOTAL**	38	100%

SBI = serious bacterial infection; UTI = urinary tract infection.

**Table 3:** Comparison of hematological laboratory parameters between the SBI and non-SBI Groups.

	SBI (N = 38)	Non-SBI (N = 33)	*p* *
**RBC (×10^12^/L)**	4.14 (3.78, 4.96)	4.46 (3.95, 4.77)	0.734
**HCT (L/L)**	0.404 (0.37, 0.46)	0.425 (0.39, 0.47)	0.360
**HGB (g/L)**	139 (127, 164.75)	148 (132, 163)	0.450
**WBC (×10^9^/L)**	13 (10.18, 16)	11.5 (9.6, 15)	0.209
**Lymphocytes (%)**	42.8 (26.32, 53.7)	46.3 (33, 58.1)	0.339
**Lymphocytes # (×10^9^/L)**	4.815 (3.36, 6.68)	4.9 (4.08, 6.61)	0.913
**MCH (pg)**	33.3 (32.7, 34.7)	34.3 (33.1, 35)	0.103
**MCHC (g/L)**	346.5 (337.25, 355.5)	347 (338, 352)	0.612
**MCV (fL)**	98.7 (94.9, 99.83)	99.3 (96.6, 102.3)	0.059
**ANC # (×10^9^/L)**	5.615 (2.85, 7.70)	3.22 (2.15, 5.47)	0.062
**Neutrophils (%)**	40.65 (29.33, 59.88)	32.1 (20.3, 53)	0.098
**Platelets (×10^9^/L)**	384 (323, 477.5)	372 (291, 443)	0.321

The data are presented as median and interquartile range. SBI = serious bacterial infection; RBC = red blood cell; HCT = hematocrit; HGB = hemoglobin; WBC = white blood cell; MCH = mean corpuscular hemoglobin; MCHC = mean corpuscular hemoglobin concentration; MCV = mean corpuscular volume; ANC = absolute neutrophil count; # = number; * Mann–Whitney U test.

**Table 4:** Comparison of biochemistry laboratory parameters between the SBI and non-SBI Groups.

	SBI (N = 38)	Non-SBI (N = 33)	*p* *
**CRP (mg/L)**	9.5 (2.3, 33.95)	2.2 (0.3, 9.9)	0.020
**Glucose (mmol/L)**	5.15 (4.4, 6.18)	5 (4.7, 5.45)	0.731
**Calcium (mmol/L)**	2.53 (2.45, 2.62)	2.54 (2.47, 2.61)	0.995
**Potassium (mmol/L)**	5.5 (5.1, 5.68)	5.4 (4.85, 5.65)	0.394
**Chlorides (mmol/L)**	103 (101, 104)	104 (102, 106.25)	0.177
**Creatinine (µml/L)**	28 (26, 31)	25 (23, 35)	0.856
**LDH (U/L)**	298 (260.5, 337)	290 (254.5, 353.5)	0.883
**Magnesium (mmol/L)**	0.82(0.78, 0.86)	0.81 (0.76, 0.87)	0.980
**Sodium (mmol/L)**	138 (137, 140.75)	138.5 (136.75, 140)	0.957
**Uric acid (µmol/L)**	161 (118, 184.5)	138 (123, 158)	0.431
**Urea (mmol/L)**	3.2 (2.6, 3.75)	2.65 (2.3, 3.3)	0.170

The data are presented as median and interquartile range. SBI = serious bacterial infection; CRP = C-reactive protein; LDH = lactate dehydrogenase. * Mann–Whitney U test.

**Table 5:** Distribution of clinical signs among the participants.

	SBI (N = 38)	Non-SBI (N = 33)	Total	%SBI	%Non-SBI	%Total	*p* *
**No visible focal source of infection**	19	17	36	50.0%	51.5%	50.7%	1
**Fever**	22	14	36	57.9%	42.4%	50.7%	0.288
**Tachycardia**	11	5	16	28.9%	15.2%	22.5%	0.270
**Breathing difficulties**	6	7	13	15.8%	21.2%	18.3%	0.778
**Poor general condition**	6	5	11	15.8%	15.2%	15.5%	1
**Mottled skin**	5	1	6	13.2%	3.0%	8.5%	0.270
**Irritability**	4	2	6	10.5%	6.1%	8.5%	0.805
**CRT > 3 s**	2	1	3	5.3%	3.0%	4.2%	1
**Weak cry**	1	1	2	2.6%	3.0%	2.8%	1
**Catarrhal pharynx**	0	1	1	0.0%	3.0%	1.4%	0.943
**Nasal congestion**	1	1	2	2.6%	3.0%	2.8%	1
**Nasal discharge**	0	1	1	0.0%	3.0%	1.4%	0.943

SBI = serious bacterial infection; CRT = capillary refill time. * Chi-square test.

**Table 6:** Urinalysis findings in neonates with UTI.

Parameter	N (%)
**Bacteria (abundant)**	19 (100%)
**Leukocyte esterase**	
**Negative**	2 (11%)
**1+**	0 (0%)
**2+**	1 (5%)
**3+**	16 (84%)
**Nitrites**	
**Positive**	12 (63%)
**Negative**	7 (37%)

## Data Availability

The raw data supporting the conclusions of this article will be made available by the authors on request due to ethical restrictions and the sensitive nature of patient medical information.
